# Water adsorption on surfaces of calcium aluminosilicate crystal phase of stone wool: a DFT study

**DOI:** 10.1038/s41598-024-59754-5

**Published:** 2024-04-21

**Authors:** Thi H. Ho, Nguyen-Hieu Hoang, Øivind Wilhelmsen, Thuat T. Trinh

**Affiliations:** 1 Laboratory for Computational Physics, Institute for Computational Science and Artificial Intelligence, Van Lang University, Ho Chi Minh City, 70000 Vietnam; 2 Faculty of Mechanical - Electrical and Computer Engineering, School of Technology, Van Lang University, Ho Chi Minh City, 700000 Vietnam; 3 Department of Materials and Nanotechnology, SINTEF Industry, 7034 Trondheim, Norway; 4 Porelab, Department of Chemistry, Norwegian University of Science and Technology, NTNU, 7491 Trondheim, Norway; 5 Department of Gas Technology, SINTEF Energy Research, 7465 Trondheim, Norway

**Keywords:** Electronic structure, Nanoscale materials, Structural materials

## Abstract

Stone wool is widely used as an efficient thermal insulator within the construction industry; however, its performance can be significantly impacted by the presence of water vapor. By altering the material’s characteristics and effective thermo-physical properties, water vapor can reduce overall efficacy in various environmental conditions. Therefore, understanding water adsorption on stone wool surfaces is crucial for optimizing insulation properties. Through the investigation of interaction between water molecules and calcium aluminosilicate (CAS) phase surfaces within stone wool using density functional theory (DFT), we can gain insight into underlying mechanisms governing water adsorption in these materials. This research aims to elucidate the molecular-level interaction between water molecules and CAS surfaces, which is essential for understanding fundamental properties that govern their adsorption process. Both dissociative and molecular adsorptions were investigated in this study. For molecular adsorption, the adsorption energy ranged from $-$ 84 to $-$ 113 kJ mol^-1^ depending on surface orientation. A wider range of adsorption energy ($-$ 132 to $-$ 236 kJ mol^-1^) was observed for dissociative adsorption. Molecular adsorption was energetically favored on (010) surfaces while dissociative adsorption was most favorable on (111) surfaces. This DFT study provides valuable insights into the water adsorption behavior on low index surfaces of CAS phase in stone wool, which can be useful for designing effective strategies to manage moisture-related issues in construction materials. Based on these findings, additional research on the dynamics and kinetics of water adsorption and desorption processes of this thermal isolation material is suggested.

## Introduction

Stone wool, a widely-used insulation material due to its excellent thermal and acoustic properties, is derived from volcanic rock or industrial waste materials^1,2^. The fibers are melted and spun into an insulating product that exhibits low thermal conductivity, reducing heat transfer by conduction and maintaining stable indoor temperatures. This makes stone wool insulation a popular choice for enhancing energy efficiency in buildings and industrial equipment^1,2^.

Significant advancements have been made in elucidating the microscale architecture of stone wool fiber. Stone wool is characterized by its predominantly calcium aluminosilicate (CAS) composition and remarkably high porosity, approximately 98%^3,4^. The unique properties and composition of stone wool are primarily attributed to CAS materials, which serve as the fundamental building blocks for its intricately interwoven fibers. This CAS material has been widely employed as a model system in the study of stone wool fiber structures and their properties^3–6^. The structure of CAS, particularly within the context of stone wool, has been investigated through minerals such as anorthite (CaAl_2_Si2O_8_) which serve as representative perfect crystal models for stone wool^5,6^. Recent studies by Ivanič et al. have utilized scanning electron microscopy (SEM), transmission electron microscopy (TEM) and other techniques to examine the surface morphology of stone wool fibers at both micrometer and nanometer scales^7^. Understanding the atomic-scale structure of stone wool fiber surfaces is crucial for manipulating their properties in various applications, such as insulation under ambient humidity levels.

The effect of moisture on stone wool insulation materials has attracted significant attention in the recent years. Moisture penetration can lead to a reduction in thermal performance as well as potential damage caused by corrosion or mold growth^8^. Temperature and water vapor have been shown to negatively affect the insulating properties of stone wool, causing a decrease in overall thermal efficiency. Comparative performance studies between stone wool and other types of insulation materials like hemp indicate that there may be some differences in their resistance to the formation of liquid water^9^. Further research has also highlighted the impact of water on the mineral wool insulations’ durability and its thermal and mechanical performance, with moisture potentially leading to a 40% reduction in the thermal resistance under real-life conditions due to various factors such as spoiling and production mistakes within the stone wool materials^10^. Another study^11^ investigated frost formation and condensation in stone-wool insulations exposed to a temperature field ranging from +20^∘^C to -20^∘^C with air on the warm side saturated with moisture. Frost accumulation was observed in parts of the specimen facing cold air, while condense formation occurred in the part facing the warm humid air^11^. With these considerations, moisture can have undesirable effects on both the thermal performance and durability of stone wool insulation materials. Understanding water vapor adsorption on stone wool surfaces is essential for optimizing their resilience against moisture-related degradation while minimizing environmental impacts associated with traditional mineral fibers^12^.

The use of computational approach for investigating the electronic structure of molecules and solids, has played an essential role in understanding the properties and behaviors of amorphous CAS materials. For example, Benoit et al.^13^ also employed DFT calculations to study the structural and electronic properties of an amorphous calcium aluminosilicate glass (CAS). The authors found that the calculated 2D-3QMAS NMR spectrum was in good agreement with experimental data, providing further validation of their CAS glass model and highlighting the utility of DFT methods for studying disordered materials such as glasses^13^. Zheng et al.^14^ investigated the micro-structure and mechanical properties of calcium aluminosilicate hydrate (C-A-S-H), a key component in cementitious materials. The authors^14^ used first-principles modeling and simulation to reveal that aluminum substitution induced interfacial strengthening in C-A-S-H, which led to an enhancement of its bulk and shear moduli. More recently, Mahadevan et al.^15^ developed a reactive potential framework for simulating dry and hydrated calcium aluminosilicate glasses by force matching and refinement, making it suitable for studying various technological applications involving these materials.

Several studies^16–20^ have been conducted using force fields to model various aspects of calcium aluminosilicate materials. For example, Kalinichev et al.^16^ used molecular dynamics modeling to study chloride binding to the surfaces of different material such as portlandite, tobermorite, and ettringite. Their results provided insight into the structure at and near the solution/solid interfaces and the molecular mechanisms of adsorption of aqueous chloride ions on portlandite surfaces. Yang et al.^17^ studied the structure, dynamics, and mechanical properties of cross-linked C-A-S-H using reactive force field molecular dynamics simulations. They found that incorporating aluminate species into C-A-S-H gels can enhance the crystalline order, improve the connectivity of Q species, and stabilize interlayer hydrogen bond connections^17^.

To the best of our knowledge, no study has been conducted utilizing advanced computational approaches to investigate the adsorption of water molecules onto CAS crystal phases in stone wool. This work employs density functional theory (DFT) simulations to elucidate the underlying mechanisms governing the adsorption process between water molecules and CAS surfaces at the molecular level in order to bridge this gap and gain valuable insights into moisture-related issues affecting construction materials. We aim to provide knowledge that can be used to increase insulation efficacy under diverse environmental situations by understanding how water vapor may drastically affect stone wool characteristics through alterations to structure and overall performance by unraveling these interactions^12^.

## Results

### Bulk structure

In this section, we present our findings from the optimization of the bulk structure of the CAS model, comparing it to available experimental data^5,6^. The primary objective is to evaluate the accuracy of our computational approach by assessing its ability to reproduce known crystal structures and properties. To achieve this goal, we have performed a comprehensive comparison between DFT-calculated crystal parameters and experimental values for the CAS phase, as reported in this study.

Table 1 presents a detailed comparison between the DFT calculated crystal parameters and experimental values for the CAS phase^5,6^. The results highlight several key dimensions of the crystal structure, including lattice parameters a, b, c; angles and overall volume. By providing these values, we aim to facilitate an in-depth analysis of the differences between theoretical predictions and empirical observations.

**Table 1:** Comparison between DFT calculated crystal parameters and experimental values for the CAS phase.

Parameter	Exp.^5,6^	PW91 (This work)	PBE_sol_ (This work)	PBE (This work)
a (Å)	8.173	8.074 ($-$ 1.2%)	8.054 ($-$ 1.5%)	8.071 ($-$ 1.2%)
b (Å)	12.869	13.047 (1.4%)	13.025 (1.2%)	13.038 (1.3%)
c (Å)	14.165	14.096 ($-$ 0.5%)	14.063 ($-$ 0.7%)	14.088 ($-$ 0.5%)
$\alpha$ (^∘^)	93.113	94.446 (1.4%)	94.301 (1.3%)	94.363 (1.3%)
$\beta$ (^∘^)	115.913	116.718 (0.7%)	116.705 (0.7%)	116.750 (0.7%)
$\gamma$ (^∘^)	91.261	92.216 (1.0%)	92.460 (1.3%)	92.270 (1.1%)
Volume (Å^3^)	1336.3	1317.7 ($-$ 1.4%)	1309.2 ($-$ 2.0%)	1315.5 ($-$ 1.6%)

Experimental data^5,6^ are provided for comparison, with values in parentheses indicating the relative deviation from the experimental data.

We found that the Generalized Gradient Approximation (GGA) functionals PW91, PBE_sol_, and PBE yield similar results for lattice constants and volume of the bulk structure of CAS, with a relative deviation of less than 2% compared to the experimental data^5,6^ (see Table 1). In contrast, the Local Density Approximation (LDA) approach (presented in the Supplementary Information) exhibits a higher deviation of approximately 4%. This result confirms that the GGA functionals provide a more accurate description of material properties compared to the LDA functionals.

### CAS surfaces

**Figure 1:**
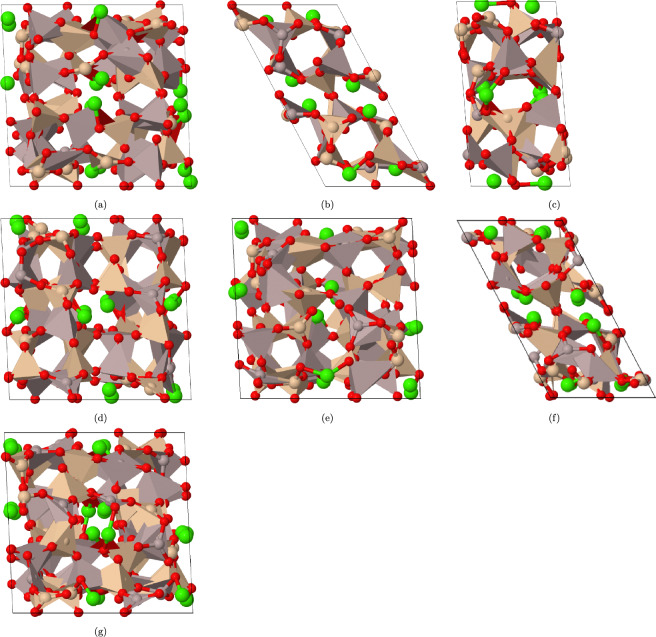
Top view of the most stable CAS surfaces. The surface index is in order (100), (010), (001), (110), (101), (011), and (111). The color of Ca, Si, Al and O atoms are represented by green, yellow, brown and red, respectively. Note that some Si atoms are situated inside SiO_4_ tetrahedral.

From the bulk crystal structure, we have generated CAS surfaces as described in Models and Methods section. To ensure that our analysis focuses on the most stable surface, we have performed geometry optimization and calculated the surface energy for each of these surfaces. The results from this optimization process are presented in Fig. 1, where the most stable surface at each orientation is plotted.

**Table 2:** Surface energies ($\gamma$ in J m^-2^) and average Bader charge (e) of different atom types of the CAS surfaces.

Surface index	$\gamma$	Ca	Si	Al	O
(1 0 0)	1.89	1.46	2.30	2.10	$-$ 1.28
(0 1 0)	1.43	1.47	2.46	2.10	$-$ 1.32
(0 0 1)	1.19	1.50	2.34	2.03	$-$ 1.28
(1 1 0)	1.13	1.50	2.47	2.10	$-$ 1.33
(1 0 1)	1.14	1.46	2.44	2.09	$-$ 1.31
(0 1 1)	1.53	1.48	2.48	2.11	$-$ 1.33
(1 1 1)	1.53	1.47	2.47	2.10	$-$ 1.33

Table 2 presents a comparison of surface energies ($\gamma$ in J m^-2^) for different low-index surfaces of the CAS phase in stone wool, along with the average Bader charge of various atom types (Ca, Si, Al, O). The surface energy values range from 1.13 to 1.89 J/m^2^. We observed that the lowest surface energy is associated with the (110) and (101) surfaces, while the highest surface energy is found for the (100) surface.

The higher Bader charge of the Si atom compared to the Al atom (see Table 2) can be attributed to its larger atomic size and stronger electrostatic interactions with surrounding atoms due to its lower electronegativity. On the other hand, Ca, being an alkaline earth metal with a larger ionic radius than aluminum, may exhibit weaker electrostatic forces resulting in relatively lower Bader charges compared to both Si and Al. The formal charges of metal atom are Si^4+^, Al^3+^, and Ca^2+^. In solid-state compounds like the CAS phase found in stone wool, these formal charges may be modified due to interactions between neighboring atoms and potential redistribution of electron density. However, despite such modifications, it is reasonable to expect that the overall trend observed through Bader charge analysis (Si > Al > Ca) still reflects the inherent electronegativity differences among Si, Al, and Ca elements.

Furthermore, as seen in Fig. S2 of the SI, it should be noted that there is no significant correlation between surface energy and the average Bader charges for individual atom types (Ca, Si, Al, or O). This suggests that the variations in surface energies across different low-index surfaces can primarily be attributed to differences in their atomic arrangements rather than the average charge distributions inside the slab.

**Figure 2:**
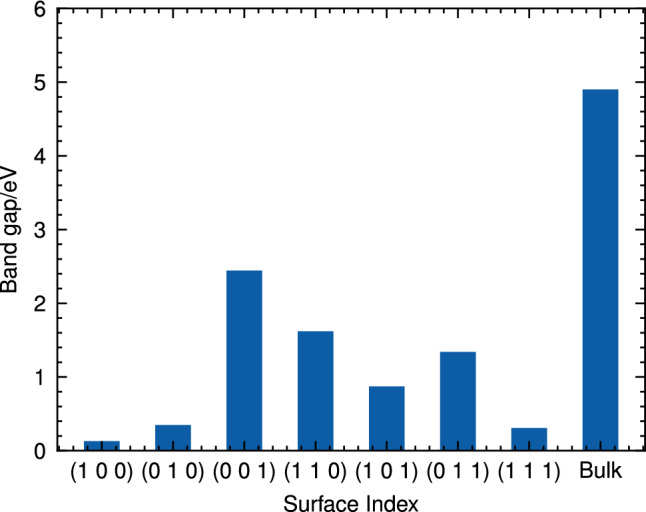
Band gap of different CAS surfaces.

Figure 2 illustrates the band gaps of CAS surfaces and bulk materials, highlighting a striking difference between them. The surface band gaps range from 0.3 to 2.5 eV, significantly lower than that of the bulk (4.9 eV). Previous research on copper oxide (Cu_2_O) surfaces has also demonstrated a similar trend in band gap reduction compared to the bulk^21^. It was also found that the number of surface layers directly affects the band gap^21^. This observation is consistent with our findings for CAS materials, suggesting that the reduction in band gap at surfaces may be a common phenomenon across different materials systems.

### Molecular adsorption of water

After determining the most stable CAS surfaces, the interaction between water molecules and these surfaces was studied at the molecular level by considering various water-molecule orientations on CAS surfaces. To ensure a thorough understanding of water adsorption behavior, we generated random adsorption sites for each orientation and optimized geometry to find the most stable configurations. The initial separation between the oxygen atom of the water molecule and the surface was established at a distance of 3.0 Å. Molecular adsorption implies that, subsequent to the adsorption process, the water molecule remained intact and did not undergo dissociation. In this context, molecular adsorption signifies the preservation of the water molecule’s structural integrity throughout its interaction with the surface, without breaking into separate components.

**Figure 3:**
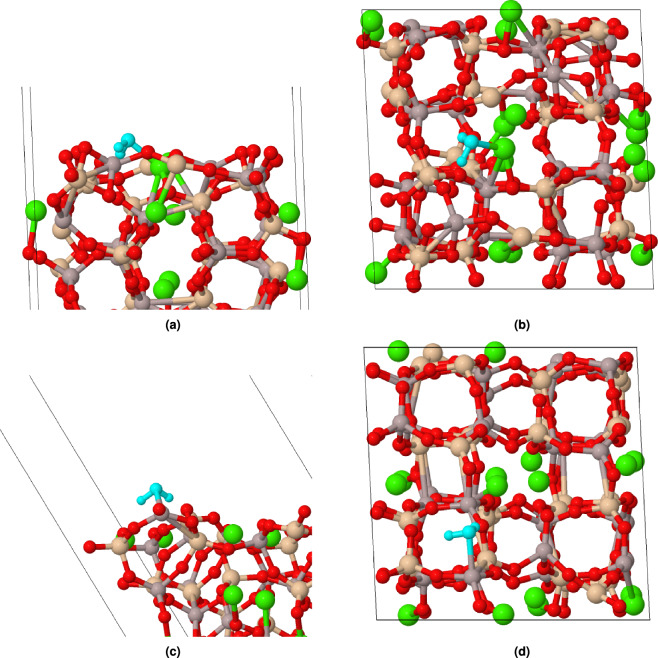
Molecular adsorption of water on CAS surfaces: (**a**) side view of (100), (**b**) top view of (100), (**c**) side view of (110) and (**d**) top view of (110). The water molecule is shown in a light blue color.

Figure 3 presents snapshots of side view and top view example of water adsorption on both (100) and (110) surfaces, illustrating how the preferred adsorption sites differ between these two CAS surfaces. In the case of the (100) surface, the water molecule is found to be in close proximity to a Ca atom, indicating that this metal ion serves as the favored adsorption site for water molecules on this particular surface. On the other hand, in the case of molecular adsorption on the (110) surface, the water molecule is positioned closer to an Al atom. This observation suggests that metal atoms play a crucial role in determining the most favorable adsorption sites for water molecules on CAS surfaces.

**Figure 4:**
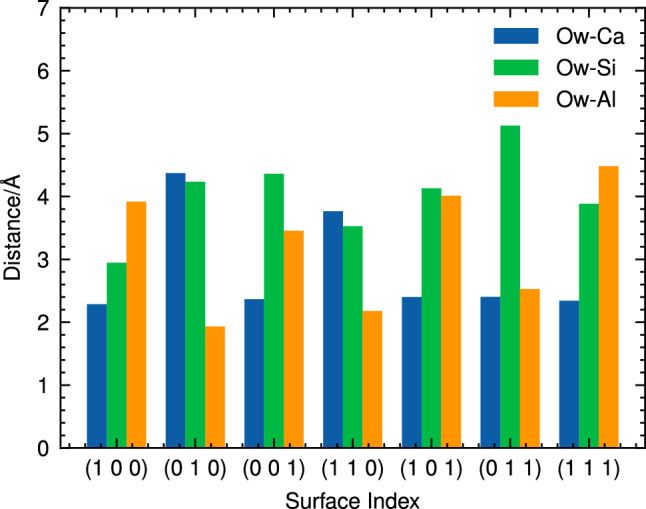
Distance between water oxygen atom (Ow) and metal sites in molecular adsorption.

Figure 4 illustrates the distance between the oxygen atom of the adsorbed water molecule (O${\textrm{w}}$) and various metal sites on CAS surfaces for molecular adsorption. As shown in this figure, the close contacts observed during molecular adsorption involves an interaction between O${\textrm{w}}$ and either Ca or Al atoms, with average distances of approximately 2.2 Å and 1.9 Å, respectively. Interestingly, no close contact was found between the oxygen atom of the water molecule and Si sites in any orientation considered during this study. It might come from the fact that the Si atoms predominantly bond with neighboring oxygen atoms to form tetrahedral SiO_4_ units. The observation that water oxygen exhibits a closer proximity to Ca and Al sites aligns well with the findings from other studies^22,23^.

**Figure 5:**
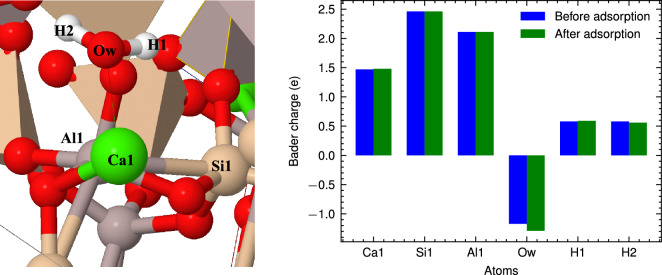
Atomic labeling of water molecular adsorption on the (100) CAS surface (on the left) and Bader charge analysis of selected atoms before and after water adsorption (on the right).

To gain a deeper understanding of how water adsorption affects the atomic charge of the surfaces, we have calculated Bader charges both before and after the molecular adsorption. For instance, Fig. 5 displays the Bader charge analysis for selected metal and water atoms before and after adsorption. We observed that electron transfer among the bonding atoms following the adsorption process. The Bader charge of Ca atom increases slightly, signifying the loss of electrons due to their interaction with the adsorbed water molecule. In contrast, the Bader charge of the Ow atom has changed from $-$1.17 e to $-$1.29 e, indicating that Ow gains 0.13 e upon adsorption. The results showed that there is an electron transfer from surface atoms to water molecules during the adsorption process on all CAS surfaces. Interestingly, this electron transfer was found to be relatively small (approximately 0.1 e), which is consistent with a previous DFT study on water adsorption on calcium silicate surfaces^23^.

### Dissociative adsorption of water

In addition to investigating molecular adsorption, we also examined the dissociative adsorption of water molecules. During this process, the incoming water molecule undergoes a structural transformation in which it splits into hydroxyl (–OH) and hydrogen (–H) species. These reactive species then bind to the surface atoms through covalent bonds, establishing stable interactions with the CAS structure.

**Figure 6:**
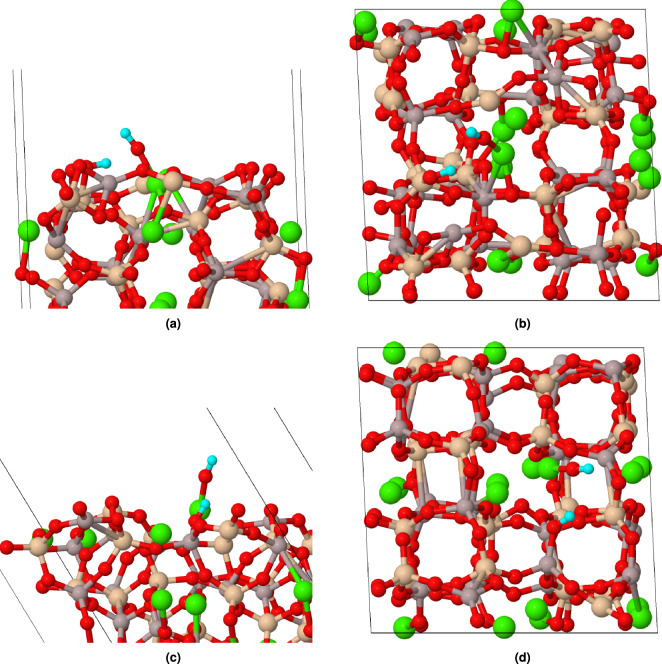
Dissociative adsorption of water on CAS surfaces: (**a**) side view of (100), (**b**) top view of (100), (**c**) side view of (110) and (**d**) top view of (110). The water molecule is shown in a light blue color.

Figure 6 presents a side view and top view example of dissociative adsorption for the (100) and (110) surfaces. This figure illustrates how the preferred adsorption sites differ between these two low-index surfaces when compared to their molecular counterparts. In both surfaces, the water molecule is found to be in close proximity to a Ca atom (around 2.2 Å) during dissociative adsorption, indicating that this Ca metal ion serves as the favored adsorption site for water molecules on these particular surfaces. It is interesting to note that when interacting with the (110) surface through dissociative adsorption, the water molecule is not positioned closer to an Al atom compared to its molecular counterpart. This observation suggests that although Al atoms play a crucial role in determining the most favorable adsorption sites for molecular adsorption, their influence may be less pronounced under dissociative conditions.

**Figure 7:**
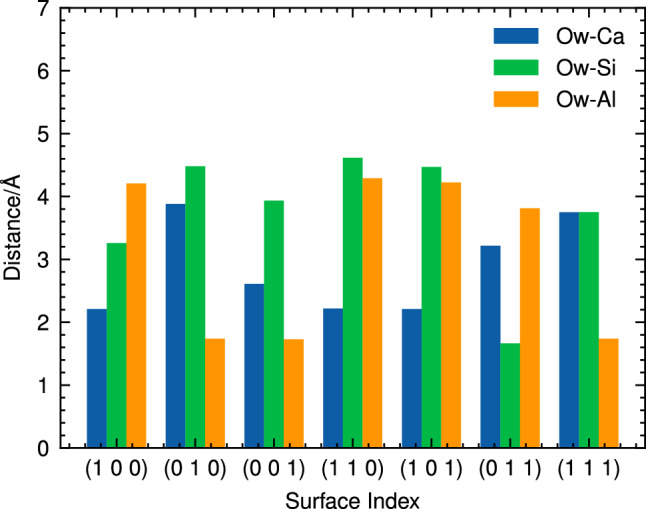
Distance between water oxygen atom (Ow) and metal sites in dissociative adsorption.

The distance between the O_w_ and various metal sites on other CAS surfaces was also analysed and presented in Fig. 7. The results reveal a more diverse range of Ow-metal distances during dissociative adsorption compared to molecular adsorption. Notably, strong interactions were observed between O_w_ and aluminum atoms on (010), (001), and (111) surfaces, while short Ow-Si distances were found exclusively on the (011) surface.

### Adsorption energy

The calculation of adsorption energies for both molecular and dissociative adsorption processes is essential for understanding the fundamental properties that govern their interactions with CAS phases under various environmental conditions. By comparing the values of these adsorption energies across different surface types, we can gain valuable insights into the dynamics and kinetics of water adsorption processes on similar material systems. This information could be potentially useful for designing effective strategies to manage moisture-related issues and enhance insulation performance under diverse construction applications.

**Table 3:** Adsorption energy (kJ mol^-1^) of molecular (M) and dissociative (D) water on the CAS surfaces.

Surface index	$E_{\text {ad}}$ (M)	$E_{\text {ad}}$ (D)
(1 0 0)	$-$ 99	$-$ 149
(0 1 0)	$-$ 113	$-$ 196
(0 0 1)	$-$ 85	$-$ 141
(1 1 0)	$-$ 107	$-$ 148
(1 0 1)	$-$ 84	$-$ 132
(0 1 1)	$-$ 77	$-$ 211
(1 1 1)	$-$ 85	$-$ 236

Table 3 presents an overview of adsorption energies for water molecule on different CAS surfaces. The range of molecular adsorption energies $E_{\text {ad}}$ (M) is from $-$ 77 to $-$ 113 kJ mol^-1^. While the value for dissociative adsorption was almost double that of molecular adsorption. The range of $E_{\text {ad}}$ (D) is from $-$ 132 to $-$ 236 kJ mol^-1^. The lowest dissociative adsorption energy is observed on the (101) surface with a value of $-$ 132 kJ mol^-1^, while the highest dissociative adsorption energy is found on the (111) surface with a value of $-$ 236 kJ mol^-1^.

An interesting observation is that the surface indices associated with the highest and lowest adsorption energies for molecular and dissociative water adsorption are not the same. Specifically, the (010) surface stands out with the highest for molecular adsorption, indicating a strong affinity for intact water molecules, while the (111) surface exhibits the highest energy for dissociative adsorption, suggesting a pronounced favorability for the dissociation process. The specific arrangement of atoms on the (111) surface may facilitate the breaking of the O–H bond in the water molecule, enabling the formation of stable covalent bonds between hydroxyl (–OH) and hydrogen (–H) species and the surface atoms.

Molecular adsorption generally exhibits lower adsorption energies compared to their dissociative counterparts, suggesting that water molecules are more likely to undergo dissociation when interacting with certain low-index surfaces of CAS phases. For instance, the (011) and (111) surfaces demonstrate significantly higher adsorption energy values for dissociative water molecules ($-$ 211 kJ mol^-1^ and $-$ 236 kJ mol^-1^, respectively) than their molecular counterparts ($-$ 77 kJ mol^-1^ and $-$ 85 kJ mol^-1^). This trend is consistent with water adsorption on other silicate surfaces from the literature^22,23^.

### Density of states and charge density difference

**Figure 8:**
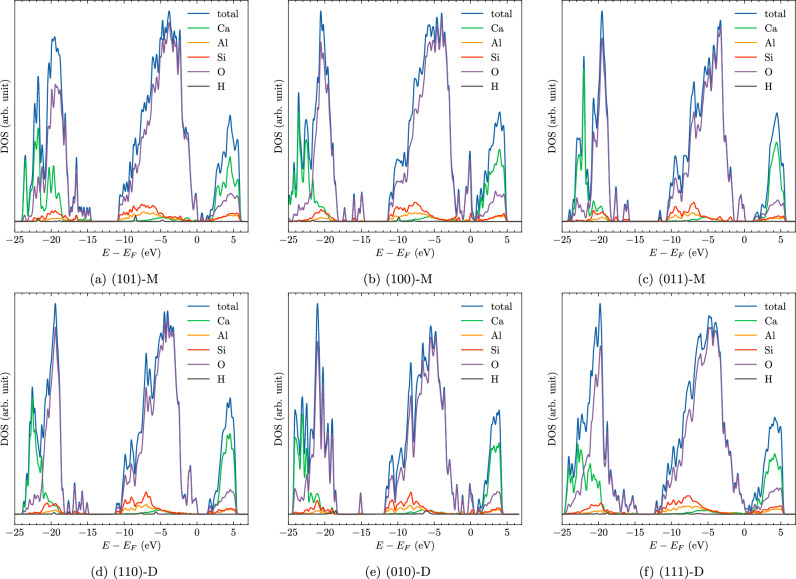
Total Density of States (DOS) with atomic contributions of water molecular (M) and dissociative (D) adsorption on selected CAS surfaces.

The density of states (DOS) for water adsorption on CAS surfaces was analyzed to gain deeper insights into their electronic structure. Figure 8 displays the total DOS, with contributions from each atom type: Ca, Si, Al, O, and H. The x-axis shows the energy of states, and the y-axis denotes the DOS. The Fermi level (E_F_) was set as a reference. It is evident that both oxygen and calcium atoms contribute significantly to the valence region of the total DOS. These contributions suggest that the atomic species are primarily involved in the formation of electronic states within the energy range associated with valence electrons. Interestingly, while calcium atoms exhibit a dominant contribution to the conductive region of the DOS, the limited contribution from hydrogen atoms indicates that hydrogen plays a lesser role in determining the DOS of CAS surfaces. The contribution of each atoms type to the total DOS of surfaces is very similar to that of the CAS bulk^24^.

In order to shed light on the bonding mechanism between water molecules and CAS surfaces, we analyzed the partial density of states (PDOS) of atoms involved in the bonding formations. Figure S3 of the SI provides a detailed illustration of the bonding associated with the water oxygen Ow atom during both molecular and dissociative adsorption on selected CAS surfaces. The Ow 2p orbital typically overlaps with the closest metal site, such as Ca 4s and Ca 3p orbitals, in valence bonds. However, this overlap is generally less significant than the bonding between the metal site and the oxygen atoms within the CAS bulk structure. This observation indicates that the bonding between the metal site and Ow atom is considerably weaker compared to the bonding between the metal site and the meta-Oxygen in the CAS bulk.

The findings from the DOS and PDOS analyses for water adsorption on CAS surfaces exhibit striking similarities to those reported in the literature for water adsorption on calcium silicate surfaces^22,23^. This notable resemblance suggests that CAS and calcium silicate systems share common trends and characteristics in their adsorption behavior, which can be attributed to their shared constituent elements. While calcium silicate surfaces lack aluminum atoms, CAS surfaces incorporate aluminum in their composition. In certain dissociative adsorption scenarios on CAS surfaces, such as those on the (010) and (111) planes, the bonding between the Ow atom and the Al site is observed instead of the Ca site. Nonetheless, the PDOS for both Al-Ow and Ca-Ow bonding exhibit similar trends (Fig. S3). This suggests that the presence of aluminum in CAS surfaces can lead to adsorption behavior that is qualitatively comparable to that of calcium silicate systems.

**Figure 9:**
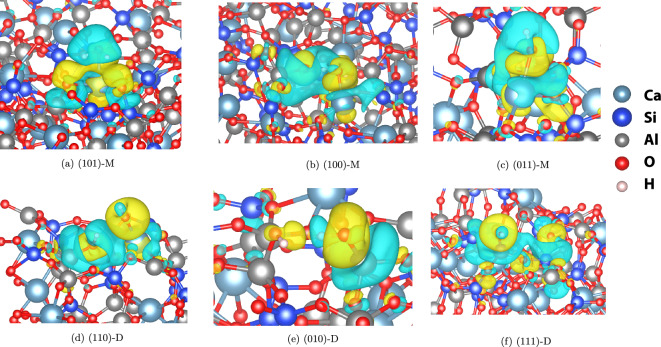
Charge density difference (CDD) of water molecular (M) and dissociative (D) adsorption on selected CAS surfaces. Gain (deficit) of charge is indicated by color yellow (cyan).

Figure 9 illustrates the charge density difference (CDD) for water adsorption on select CAS surfaces. In this visualization, areas of charge gain are depicted in yellow, while regions of charge deficit appear in cyan. For the sake of clarity, only a portion of the system surrounding the water molecule is plotted. There is a charge transfer from the CAS surfaces to the Ow atom of the adsorbed water molecule in all cases. This observation is consistent with previous findings^22,23^ and highlights the significant role that charge transfer plays in determining the bonding interactions between water molecules and CAS surfaces.

## Discussion

The investigation of water adsorption behavior on CAS surfaces in stone wool insulation materials revealed that both molecular and dissociative modes of interaction can occur when a water molecule interacts with these surfaces. The results of the study suggest that water molecules do not exhibit very strong favorable interactions with specific low-index surfaces when considering molecular adsorption. The difference between the lowest and highest adsorption energy values for molecular water adsorption is only 36 kJ mol^-1^, indicating a relatively weak preference for certain surface orientations. However, when examining dissociative adsorption, we observed a much larger difference in adsorption energies between the lowest and highest value (104 kJ mol^-1^). This indicates a stronger favorable interaction between the water molecule and specific CAS surfaces under dissociative conditions. This finding highlights the importance of considering both adsorption mechanisms when studying water interaction with construction materials like stone wool, as it may have significant implications for optimizing insulation performance under various environmental conditions.

It’s noteworthy that while the data provides valuable insights into the adsorption energies, the need for further exploration into the activation barrier for dissociating water on CAS surfaces. The activation barrier represents the energy required to initiate the process, and understanding this barrier for dissociative adsorption would provide critical information about the feasibility and kinetics of this process.

Water adsorption and desorption on CAS surfaces can have distinct characteristics that are primarily influenced by molecular (M) and dissociative (D) adsorption mechanisms. The different energetics and interactions associated with intact molecular water and dissociated water species cause asymmetry in adsorption and desorption behaviors. The process of molecular adsorption, in which intact water molecules adhere to the surface without dissociation, is less energetically demanding. Dissociative adsorption, on the other hand, involves the separation of water molecules upon interaction with the CAS surface. As demonstrated by this study, this process necessitates a higher energy input. As a result, desorption of dissociated water species may require breaking through a higher energy barrier.

The analysis of DOS, PDOS, and CDD provides valuable insights into the electronic structure, bonding interactions, and adsorption properties of water molecules on CAS surfaces. We find that the total DOS is primarily influenced by the oxygen and calcium atoms, with smaller contribution from other elements. The PDOS reveals that the bonding between the metal site and bulk oxygen atoms is stronger than the interaction between the metal site and the Ow atom of the water molecule. The CDD visualizations demonstrate that a charge transfer occurs from the CAS surface to the Ow atoms of the adsorbed water molecules. This finding aligns with the previous observations on calcium silicate surfaces^22,23^, indicating a consistent charge transfer mechanism between the surface and adsorbent.

To the best of our knowledge, the experimental data of adsorption energy of water on CAS surfaces has not been reported. Thus, we compare the results of this work with previously published DFT calculations of water adsorption on dicalcium silicate (C_2_S) and tricalcium silicate (C_3_S) surfaces^23^. Our DFT calculations reveal that the molecular adsorption of water on CAS surfaces exhibits a range of adsorption energies from $-$ 77 to $-$ 113 kJ/mol. This is in close agreement with the molecular adsorption energy ranges reported for water on C_2_S ($-$ 73 to $-$ 116 kJ/mol) and C_3_S ($-$ 71 to $-$ 129 kJ/mol) surfaces^23^. For the dissociative adsorption, we find that CAS surfaces exhibit a range of adsorption energies from $-$ 141 to $-$ 236 kJ/mol, which is also comparable to the values reported for water on C_3_S surfaces ($-$ 157 to $-$ 313 kJ/mol). These observations indicate that water adsorption on CAS surfaces shares similarities with C_3_S surfaces in both molecular and dissociative adsorption regimes.

## Conclusions

The results from systematic DFT calculations of water adsorption behavior on low-index surfaces of calcium aluminosilicate (CAS) phases within stone wool insulation materials have been presented and discussed. The findings has revealed a dual interaction mechanism involving both molecular and dissociative modes of interaction.

The observed range of adsorption energies emphasizes these distinctions even more. For molecular adsorption, the range spans from $-$ 84 to $-$ 113 kJ mol^-1^, indicating the subtle variations in interaction strengths across different CAS surfaces. In contrast, the wider range of $-$ 132 to $-$ 236 kJ mol^-1^ for dissociative adsorption underlines the significantly divergent and stronger interactions occurring in this mode. Molecular adsorption is most favorable on the (010) surface, while dissociative adsorption is preferred on the (111) surface.

The calculations revealed an electron transfer mechanism from metal atoms within the CAS surfaces to oxygen atoms within the adsorbed water species. We observed that preferred adsorption sites for water molecules depend on the specific low-index surface being considered. For instance, Ca atoms served as favored adsorption sites on the (100) surface while Al atoms played a crucial role in determining the most favorable adsorption sites on the (110) surface. This highlights the need to consider surface-specific interactions for tackling moisture issues in stone wool insulation material.

Taking this work as a starting point, future research could employ simulations like grand canonical Monte Carlo (GCMC) or Molecular Dynamics to study adsorption/desorption processes at diffrent temperatures and presures. Additionally, investigating factors such as surface defects or impurities within CAS materials can enrich our understanding of water-surface interactions and aid in developing strategies for managing moisture issues and optimizing insulation performance under diverse conditions.

## Models and methods

**Figure 10:**
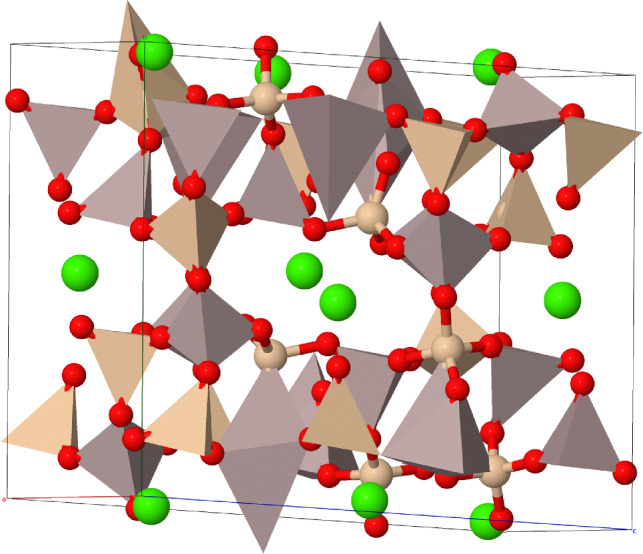
Unit cell of CAS model (Anorthite). The color of Ca, Si, Al and O atoms are represented by green, yellow, brown and red, respectively.

DFT calculations were performed using the Vienna Ab initio Simulation Package (VASP) software^25,26^. The Perdew-Burke-Ernzerhof (PBE) exchange-correlation functional was employed to describe the electronic structure and energetics of the systems under investigation. The PBE functional is one of the most common DFT methods in solid state calculation and was previously applied to study the properties of solid cement clinker phases^27,28^. A recent study by Ho et al.^24^ demonstrates the successful application of PBE functional for calculating structural, electronic, and mechanical properties of the CAS phase. In addition, PW91^29^, PBEsol^30^ and the Local Density Approximation (LDA) approach were also used to optimize the crystal structure of the bulk CAS. We found that the PBE functional is one of the suitable Generalized Gradient Approximation (GGA) functionals for calculating crystal parameters. In agreement with a recent study by Ho et al.^24^, we have chosen the PBE functional for surface and water adsorption calculations. It has been demonstrated that the inclusion of Van der Waals (VdW) corrections in DFT calculations play an important role in obtaining accurate results for silicate material^27,28,31^. We employed the Grimme 3D approach^32^ to add VdW correction in this study.

As mentioned earlier, the anorthite crystal is served as the CAS model (see Fig. 10). The initial crystal structure of anorthite was obtained from the American Mineralogist Crystal Structure Database^33^. The atomic positions and crystal parameters (3 lattice parameters a, b, c and 3 angles $\alpha$, $\beta$, $\gamma$) were relaxed using the DFT optimization algorithm implemented in VASP. The convergence criterion for energy was set to 1.0$\times$10^-6^ eV to ensure accurate calculations. The Monkhorst-Pack k-point mesh was used for Brillouin zone sampling, with a density appropriate for the specific system. A plane-wave basis set was used with an energy cutoff of 500 eV. This energy cutoff was chosen based on convergence tests to balance accuracy and computational efficiency. The structures of the phases were plotted using the VESTA^34^ and the Jmol^35^ packages. The Bader charge analysis was carried out since it has been shown to be an effective method of representing chemical reactivity^36^.

The low-index surfaces of CAS were cleaved from the optimised bulk unit cell. The in-plane crystalline periodicity was retained during the slab model construction. A vacuum thickness of 15 Å was added in the slab. All surfaces were kept stoichiometric and neutral to avoid the polarising electric field^37,38^.

No symmetry was imposed on the slab model since they might naturally have different atomic arrangements^39^. All atoms in the slab were allowed to relax while the in-plane lattice parameters were fixed. The surface relaxation was carried out until the maximum residual force on each atom converged to 0.05 eV Å^-1^. Dipole correction was applied during the surface relaxation. The slab model and Brillouin zone mesh for CAS surfaces are presented in Table 4.

**Table 4:** Slab model and Brillouin zone mesh for CAS surfaces. Slab model size (a, b, c) and number of formula unit (CaAl_2_Si_2_O8)n.

Surface index	a	b	c	n	Brillouin mesh
(1 0 0)	13.038	14.088	38.727	16	$2 \times 2 \times 1$
(0 1 0)	8.071	14.088	39.165	8	$3 \times 2 \times 1$
(0 0 1)	8.101	13.008	42.212	8	$3 \times 2 \times 1$
(1 1 0)	14.088	15.401	38.727	16	$2 \times 2 \times 1$
(1 0 1)	13.038	19.618	38.727	16	$2 \times 1 \times 1$
(0 1 1)	8.071	19.978	52.221	16	$3 \times 1 \times 1$
(1 1 1)	15.401	19.618	54.218	24	$2 \times 1 \times 1$

The surface energy $\gamma$ is an important parameter to characterise the CAS surfaces, which can be calculated as follows:

1 $$\begin{aligned} \gamma = \frac{1}{2A} \left( E_{\text {slab}} - n \cdot E_{\text {bulk}} \right) \end{aligned}$$

where $E_{\text {slab}}$ is the total energy of the slab model, n is the number of formula units contained in the slab model, $E_{\text {bulk}}$ is the total energy per formula unit of the bulk structure, and A is the surface area of the slab.

Water molecules were initially placed at random adsorption sites, including all metal atoms (Ca, Al, and Si) on the CAS surface. Following energy minimization, we report only the most stable adsorption configuration for each surface in this study. The adsorption energy $E_{\text {ad}}$ of water molecule can be calculated as follows:

2 $$\begin{aligned} E_{\text {ad}} = E_{\text {slab-water}} - \gamma \times {2A} - E_{\text {water}} \end{aligned}$$

where $E_{\text {slab-water}}$ is the total energy of the surface and water system after adsorption and $E_{\text {water}}$ is the energy of an isolated water molecule in a vacuum. A negative $E_{\text {ad}}$ represents the adsorption is energetically favored.

### Additional information

Supplementary Information (SI) is available to provide additional details of the study including the convergence test for cut-off energy, local density approximation (LDA) functional, and partial density of states (PDOS).

### Supplementary Information

Supplementary Information.

## Data Availability

The datasets generated and/or analyzed during the current study are available from the corresponding author on reasonable request.
