# Supplementary material for: Water adsorption on surfaces of calcium aluminosilicate crystal phase of stone wool: a DFT study
Source: Sci Rep. 2024 Apr 21;14:9135. doi: 10.1038/s41598-024-59754-5 (PMC11033287; doi:10.1038/s41598-024-59754-5)
Supplement: Supplementary file 1 — Supplementary Information. [file 41598_2024_59754_MOESM1_ESM.pdf]

Supporting Information: Water adsorption on surfaces of calcium aluminosilicate crystal phase of stone wool: A DFT study

Thi H. Ho,[†,‡] Nguyen-Hieu Hoang,[¶] Øivind Wilhelmsen,[§,∥] and Thuat T. Trinh[*,§]

†*Laboratory for Computational Physics, Institute for Computational Science and Artificial Intelligence, Van Lang University, Ho Chi Minh City, 700000, Vietnam*

‡*Faculty of Mechanical - Electrical and Computer Engineering, School of Technology, Van Lang University, Ho Chi Minh City, 700000, Vietnam*

¶*Department of Materials and Nanotechnology, SINTEF Industry, Trondheim, NO-7034, Norway*

§*Porelab, Department of Chemistry, Norwegian University of Science and Technology, Høgskoleringen 5, 7491-Trondheim, Norway*

∥*Department of Gas Technology, SINTEF Energy Research, Trondheim, NO-7465, Norway*

E-mail: thuat.trinh@ntnu.no

Convergence test

Figure S1 illustrates an energy convergence test, demonstrating that a cutoff value of 500 eV is adequate for obtaining accurate and reliable results in our calculations.

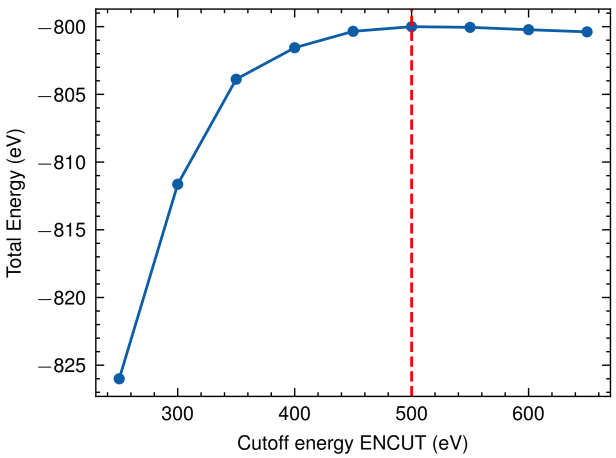

Figure S1: The total energy with respect to the cutoff energy. The ENCUT = 500 eV is sufficient.

Extra DFT calculation with LDA functional

Table S1 presents supplementary crystal parameters calculated using the Local Density Approximation (LDA) method. As expected, the LDA method yields results of lower quality compared to the Generalized Gradient Approximation (GGA). For a detailed discussion, please refer to the relevant sections of the main text.

Table S1: Comparison between LDA calculated crystal parameters and experimental values for the CAS phase. Experimental data[S1,S2] are provided for comparison, with values in parentheses indicating the relative deviation from the experimental data.

Parameter	Exp.[S1,S2]	LDA (This work)
a (Å)	8.173	7.883 (-3.6%)
b (Å)	12.869	13.081 (1.6%)
c (Å)	14.165	13.946 (-1.5%)
α (°)	93.113	95.015 (2.0%)
β (°)	115.913	115.445 (-0.4%)
γ (°)	91.261	93.044 (2.0%)
Volume (Å^3)	1336.3	1286.9 (-3.7%)

Bader charge

Figure S2 displays the average Bader charges for various atom types on the calcium aluminosilicate (CAS) surface. These values can be found in Table 2 of the main text.

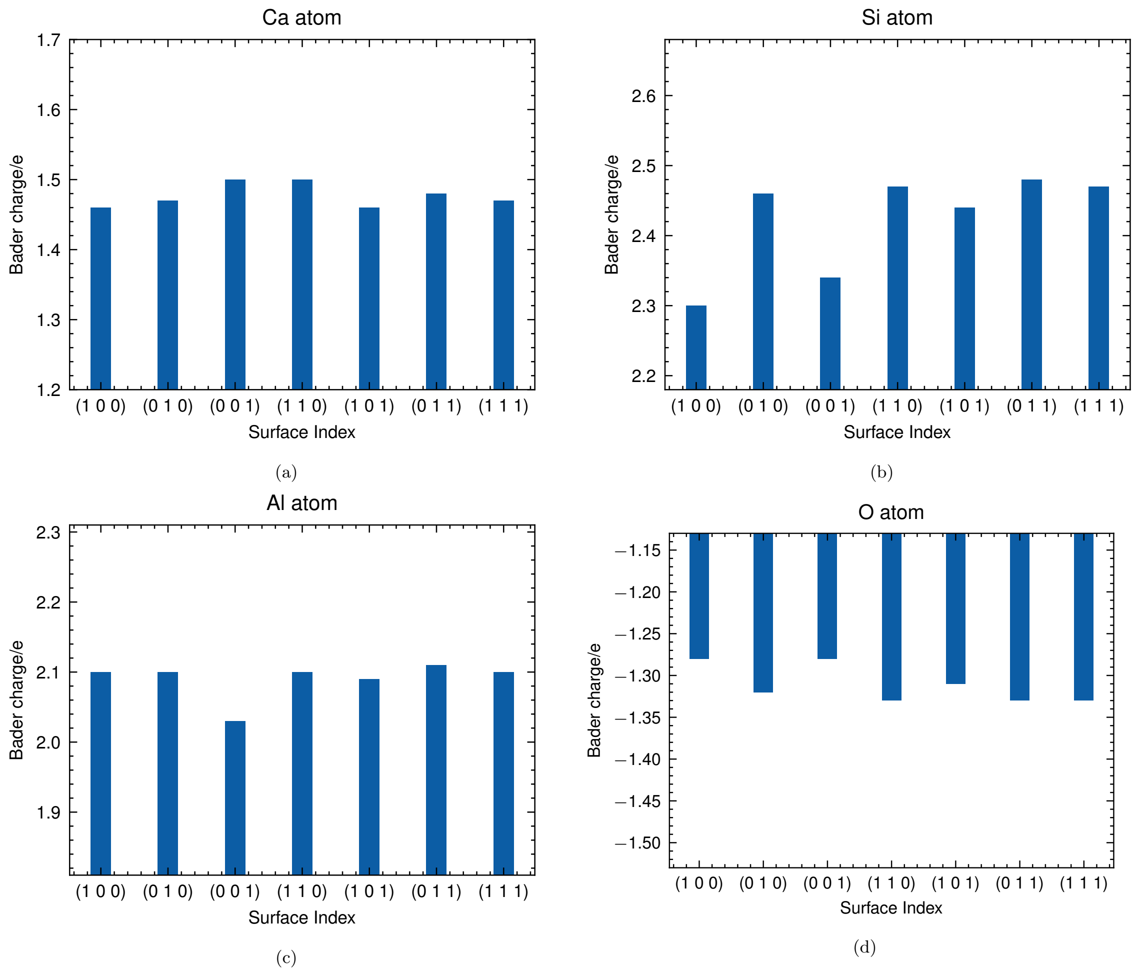

Figure S2: Average of Bader charge of different atom type of CAS surfaces.

Partial Density of States

Figure S3 shows the Partial density of states (PDOS) of the metal adsorption sites and water oxygen atoms. More discussion is presented in the main text.

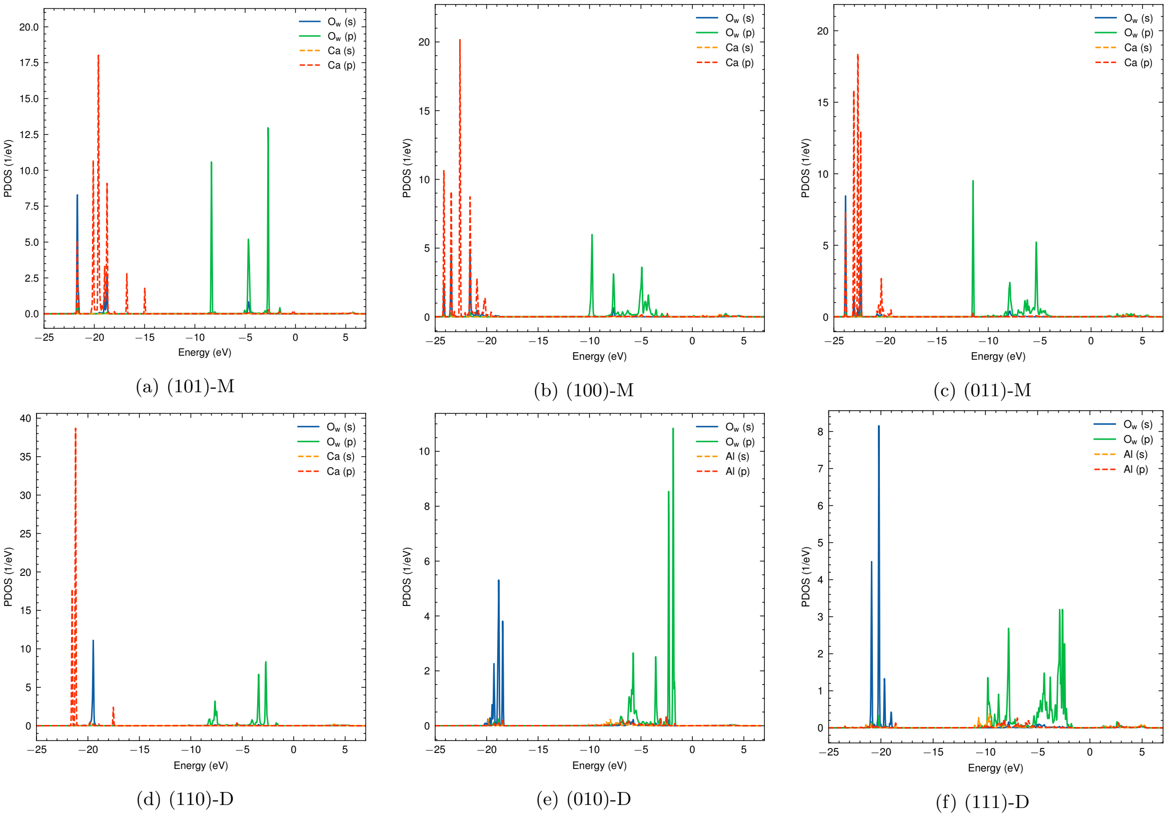

(a) (101)-M (b) (100)-M (c) (011)-M

(d) (110)-D (e) (010)-D (f) (111)-D

Figure S3: Partial density of states (PDOS) of the metal adsorption sites and water molecular (M) and dissociative (D) adsorption on selected CAS surfaces.

References

(S1) Traoré, K.; Kabré, T. S.; Blanchart, P. Gehlenite and anorthite crystallisation from kaolinite and calcite mix. *Ceram. Int.* **2003**, *29*, 377–383.

(S2) Megaw, H. D.; Kempster, C.; Radoslovich, E. The structure of anorthite, CaAl2Si2O8. II. Description and discussion. *Acta Crystallogr.* **1962**, *15*, 1017–1035.
